# Global Trends in Non‐Technical Skills Research in Dental Education: Bibliometric Review and a Curricular Case Study

**DOI:** 10.1155/ijod/8633389

**Published:** 2026-01-26

**Authors:** Tanit Arunratanothai, Supachai Chuenjitwongsa, Kanoknadda Tavedhikul, Piyamas Sumrejkanchanakij, Kausar Sadia Fakhruddin, Lakshman Samaranayake, Thanaphum Osathanon

**Affiliations:** ^1^ Dental Education Unit, Office of Academic Affairs, Faculty of Dentistry, Chulalongkorn University, Bangkok, Thailand, chula.ac.th; ^2^ Department of Biochemistry, Faculty of Dentistry, Chulalongkorn University, Bangkok, Thailand, chula.ac.th; ^3^ Department of Periodontology, Faculty of Dentistry, Chulalongkorn University, Bangkok, Thailand, chula.ac.th; ^4^ Center of Excellence for Dental Stem Cell Biology, Department of Anatomy, Faculty of Dentistry, Chulalongkorn University, Bangkok, Thailand, chula.ac.th; ^5^ UWA Dental School, University of Western Australia, Perth, Western Australia, Australia, uwa.edu.au; ^6^ Global Research Cells, Dr. D. Y. Patil Dental College and Hospital, Dr. D. Y. Patil Vidyapeeth, Pimpri, Pune, India, dpu.edu.in

**Keywords:** bibliometric analysis, communication skills, cultural competence, curriculum integration, dental education, leadership, research trends, soft skills

## Abstract

**Background:**

Non‐technical (soft) skills such as communication, professionalism, leadership and cultural competence are essential elements of patient‐centred care, yet their integration into dental curricula remains inconsistent. To our knowledge, no bibliometric study has systematically mapped these global trends, leaving gaps in understanding and research. This study aimed to (i) conduct a comprehensive bibliometric mapping of global research trends on non‐technical (soft) skills in dental education, highlighting thematic patterns, underrepresented domains and geographic disparities and (ii) demonstrate how these insights can be mapped to the curriculum, illustrated through an implementation case study from a Faculty of Dentistry in Thailand.

**Methods:**

We conducted a bibliometric analysis of English‐language articles and reviews from Scopus (upto February 9, 2025). Studies were included if they focused on non‐technical (soft) skills training or assessment for dental students, faculty or practitioners. Using VOSviewer, Microsoft Excel and GraphPad Prism, the publication trends, sources, citations, geographic distribution and keyword co‐occurrence were analysed. The quantitative findings were directly applied to contextualise a curriculum mapping exercise at the Faculty of Dentistry, Chulalongkorn University, Thailand.

**Results:**

Analysis of 546 publications revealed a 40% increase in annual output over the last decade. Research was highly concentrated in high‐income countries, led by the United States, with Africa and South America collectively contributing a meagre < 5%. Keyword analysis identified five thematic domains: i) professionalism/leadership, ii) communication skills, iii) personality/emotional intelligence, iv) education/assessment and v) behavioural sciences. Significant thematic disparities were evident, with communication skills being the dominant element while leadership, cultural competence and entrepreneurship were markedly underrepresented. Citation patterns mirrored these imbalances. The case study from Thailand served as an illustrative model demonstrating the integration of these underrepresented skills, such as leadership, conflict resolution and cultural sensitivity, across a 6‐year curriculum through diverse pedagogical and assessment strategies.

**Conclusion:**

This study provides the bibliometric map of non‐technical (soft) skills research in dental education, revealing not only growth but also profound geographic and thematic imbalances. More importantly, it moves beyond analysis to present a dual‐method framework: using global data to identify critical gaps and then providing a replicable model for curriculum intervention. This approach offers dental educators a data‐driven blueprint for designing equitable, comprehensive and contextually relevant curricula that prepare graduates for the complex demands of modern healthcare.

## 1. Introduction

Non‐technical (soft) skills in dentistry, such as communication, critical thinking, teamwork, leadership, professionalism, lifelong learning, entrepreneurship, problem‐solving, professional ethics, and moral and cultural competence and adaptability, are essential for delivering patient‐centred care and complement technical expertise [1, 2]. These interpersonal and cognitive competencies, including values, empathy and commitment, enable dental professionals to collaborate effectively and thrive in dynamic clinical settings [2]. Additionally, management‐related competencies such as conflict resolution, time and stress management, decision‐making and practice management are increasingly emphasised as core components of professional readiness [1]. Traditionally, dental curricula have focused on technical and clinical training. However, in recent years, the growing emphasis on holistic patient‐centred care has highlighted the importance of non‐technical (soft) skills [1]. This shift aligns with broader trends in healthcare, which are moving towards interprofessional collaboration and are central to achieving optimal health outcomes.

Multiple studies have highlighted the importance of non‐technical (soft) skills in dental practice. In this regard, effective dentist–patient communication fosters trust, improves patient engagement and enhances treatment adherence [3]. Similarly, interprofessional teamwork is linked to better care outcomes; however, dental students often lack a deep understanding of collaborative roles [4]. Other non‐technical (soft) skills, such as leadership and problem‐solving, are increasingly vital for navigating the complexities of modern dental practice [1].

Despite growing awareness, global research on non‐technical (soft) skills in dental education remains fragmented, thematically skewed and geographically uneven. While topics such as communication and professionalism appear well‐represented, other domains, including leadership, ethics and entrepreneurship, appear underexplored. Moreover, the contributions of specific countries, institutions and scholars have not been systematically mapped. To date, no bibliometric study has comprehensively analysed the temporal growth, thematic clusters, collaborative networks and citation patterns in the domain of non‐technical (soft) skills in dentistry. Evidently, this gap limits educators’ and policymakers’ ability to assess progress, identify research needs and align curricula with emerging evidence.

Therefore, the present study was designed to address two key objectives: first, to conduct a comprehensive bibliometric analysis of global research on non‐technical (soft) skills in dental education to identify key themes, gaps, contributors and publication trends. Next, the opportunity was taken to demonstrate how these insights can be mapped to practice through an example of curriculum integration of non‐technical (soft) skills from the Faculty of Dentistry at Chulalongkorn University, Thailand. By bridging macro‐level bibliometric findings with a specific element in curriculum development, this study offers a replicable model for advancing both scholarly understanding and educational practice.

## 2. Methodology

A literature search was conducted using the Scopus database up to February 9, 2025, as the primary source for this bibliometric study. Scopus was chosen as the primary database due to its broad coverage of peer‐reviewed literature in health sciences and education, robust citation analysis tools and inclusion of a wider range of international journals compared to alternatives. The complete search strategy is provided in Supporting Information 1: Table S1. Searches were restricted to the article title field to ensure the specificity and relevance of the identified articles. Only peer‐reviewed research and review articles published in English were included.

The studies were included if they met the following exclusion and inclusion criteria. Inclusion criteria were those that addressed non‐technical (soft) skills or personality development in dental education or practice; examined the status, competency or training of non‐technical (soft) skills or personality attributes; and described or evaluated methods, curricula or interventions related to non‐technical (soft) skills or personality. Exclusion criteria included editorials, opinion pieces, book chapters, letters, conference proceedings and abstracts; and studies that focused solely on clinical or technical skills without content related to non‐technical (soft) skills. Eligible study populations were dental students, dental faculty members or licensed dental professionals.

**Figure 1:**
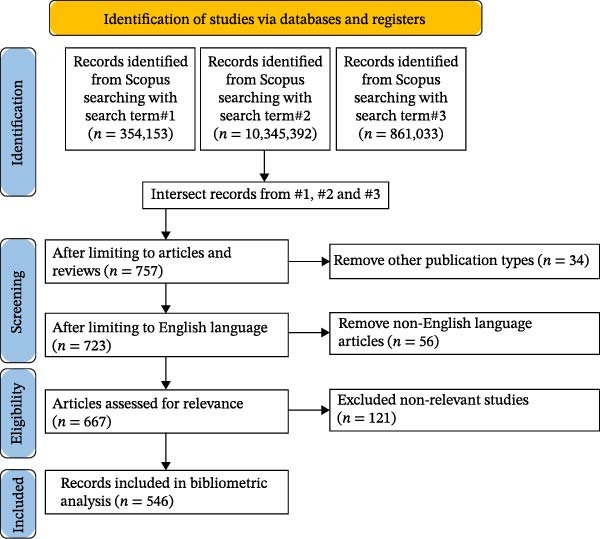
Flow chart of the process of the study selection.

Electronic data searches and analyses were conducted in accordance with the PRISMA guidelines. Upon identifying the relevant publications, these resources were subsequently exported for further examination (Figure 1). After removing duplicate publications and filtering by publication type and English language, the relevant studies were independently screened by TA and TO. A conflict in the selection was resolved by discussion between TA and TO. Non‐relevant studies, such as those not related to non‐technical (soft) skills or not in the field of dentistry, were removed. A comprehensive record was maintained documenting the number of publications, the various journals from which they originated, the corresponding citation counts, the countries of origin and the keywords associated with each publication, all meticulously organised in a CSV format to facilitate further analysis. In the present study, grey literature, trial registries, hand‐searching or citation chasing were not included in the methodology. The present study aimed to identify the landscape of publications rather than to scope current knowledge. Hence, the publications were not appraised for quality.

The software tool VOSviewer, version 16.20, developed by Leiden University [5], was employed to analyse the intricate co‐occurrence relationships among the identified publications. All keywords that occurred at least 10 times were included to construct the co‐occurrence network. The identified keywords were then manually screened by TO. To ensure their relevance to the personality and non‐technical (soft) skills for further analysis, the network map, co‐occurrence matrix and keyword cluster analysis were generated with this tool to uncover the thematic landscape and dynamic evolution of the research field. Microsoft Excel (Microsoft Corporation, Redmond, WA, USA) and GraphPad Prism (GraphPad Software, San Diego, CA, USA) were utilised to tabulate the data and generate supplementary visualisations.

Finally, to contextualise the bibliometric findings, a brief example of curriculum integration from the Faculty of Dentistry at Chulalongkorn University was included to illustrate how research trends can inform educational practice.

## 3. Results

### 3.1. Publication Trends Reveal Growing Interest in Non‐Technical (Soft) Skills

**Figure 2:**
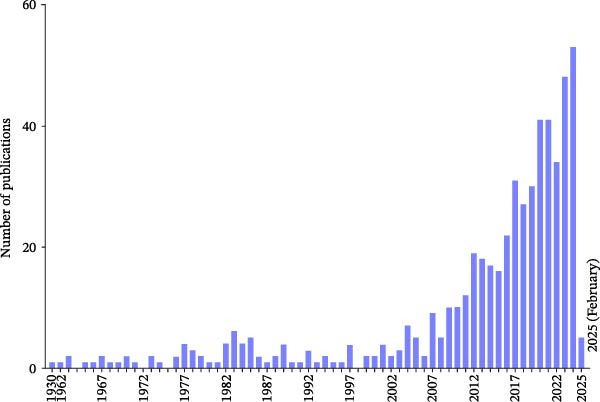
Trends in global publications on non‐technical (soft) skills in dental education (2000–2025). Annual number of publications indexed in Scopus meeting the inclusion criteria, showing a steady rise over time with a marked 40% increase in output from 2015 to 2025. This growth reflects increasing scholarly interest in integrating non‐technical competencies, such as communication, professionalism and leadership, into dental curricula worldwide.

A total of 546 publications met the inclusion criteria for bibliometric analysis (Figure 1). The publication trend demonstrated a steady growth over the study period, with a notable inflection point after 2012. Between 2015 and 2025, there was a 40% growth in annual publication output, implying growing scholarly interest in non‐technical (soft) skills in dental education (Figure 2).

Three journals accounted for ~42% of the total output. The Journal of Dental Education topped the league with 164 publications, followed by the European Journal of Dental Education with 61 publications and the British Dental Journal with 21 publications (Table 1). This concentration of publications in education‐focused journals reflects the centrality of non‐technical (soft) skills discourse to dental education scholarship.

**Table 1:** Number of publications and citations by sources.

Sources	Documents	Citations	Citation per publications
Journal of Dental Education	164	2412	14.70
European Journal of Dental Education	61	790	12.95
British Dental Journal	21	341	16.23
BMC Medical Education	13	82	6.31
BMC Oral Health	6	24	4.00
European Journal of Dentistry	5	57	11.40
Journal of the American Dental Association	5	11	2.20

### 3.2. Geographic Imbalances in Research Productivity and Collaboration Networks

Publication output was predominantly from high‐income countries (Table 2). The United States led with 148 publications (~27%), followed by the United Kingdom (56 publications) and India (40 publications). Contributions from Africa and South America collectively accounted for less than 5% of publications, highlighting a significant geographic imbalance.

**Table 2:** Top 10 publications and citations by location.

Country	Documents	Citations
United States	148	1601
United Kingdom	56	742
India	40	246
Saudi Arabia	25	217
Canada	23	375
Germany	19	241
Chile	17	229
Pakistan	18	68
Australia	15	166
Japan	14	82

Co‐authorship mapping revealed six international collaboration clusters (Figure 3A). Notably strong partnerships were observed between the United States–Chile (Cluster 1) and the UK–India–Japan (Cluster 4). In contrast, low‐resource regions demonstrated limited integration into the global network, underscoring opportunities for capacity‐building initiatives.

Figure 3Global collaboration and citation networks in non‐technical (soft) skills research within dental education. (A) International co‐authorship network illustrating six major collaboration clusters, with notable partnerships between high‐output countries, for example, the United States–Chile. (B) Cross‐country citation network, showing the influence and interconnectedness of research outputs, with the United States, United Kingdom, Canada and India occupying central positions. Visualisations generated using VOSviewer.

**Figure:**
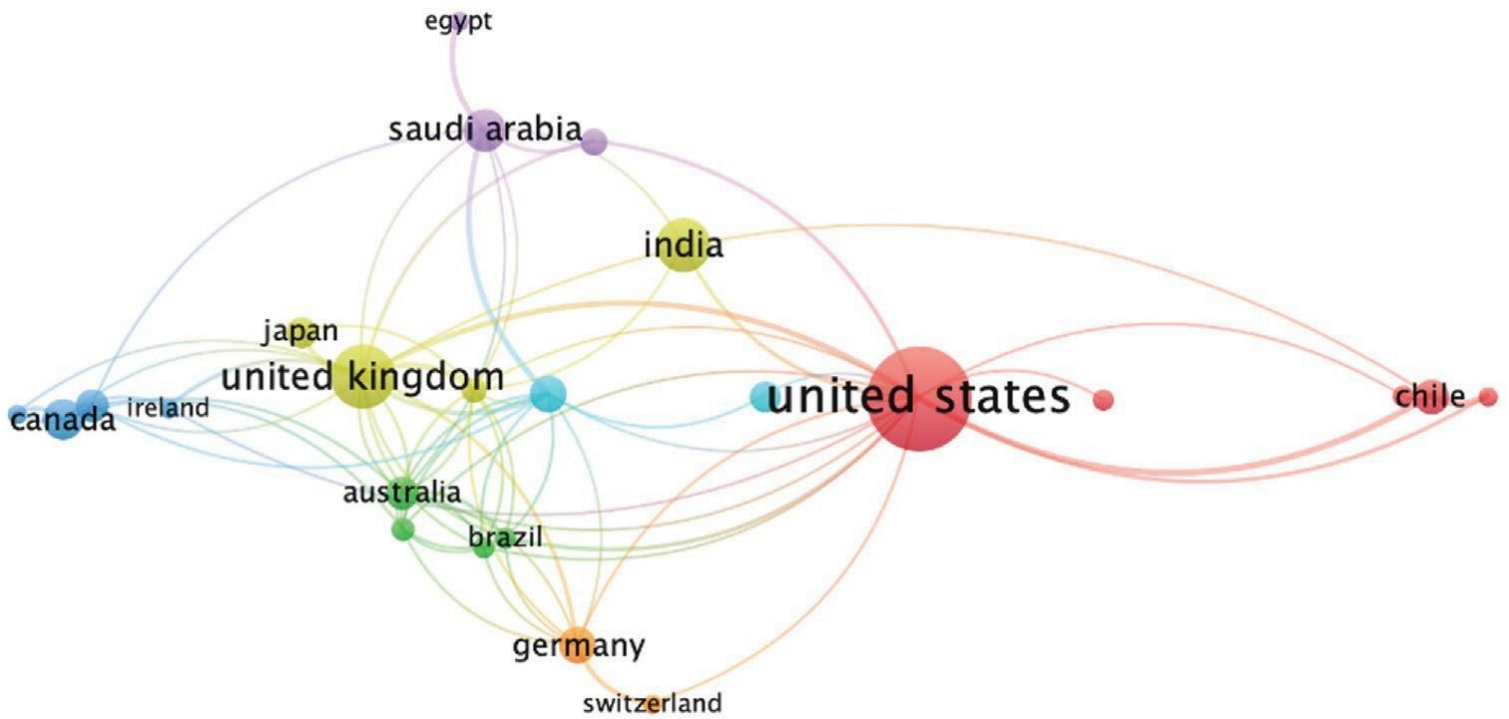
(A)

**Figure:**
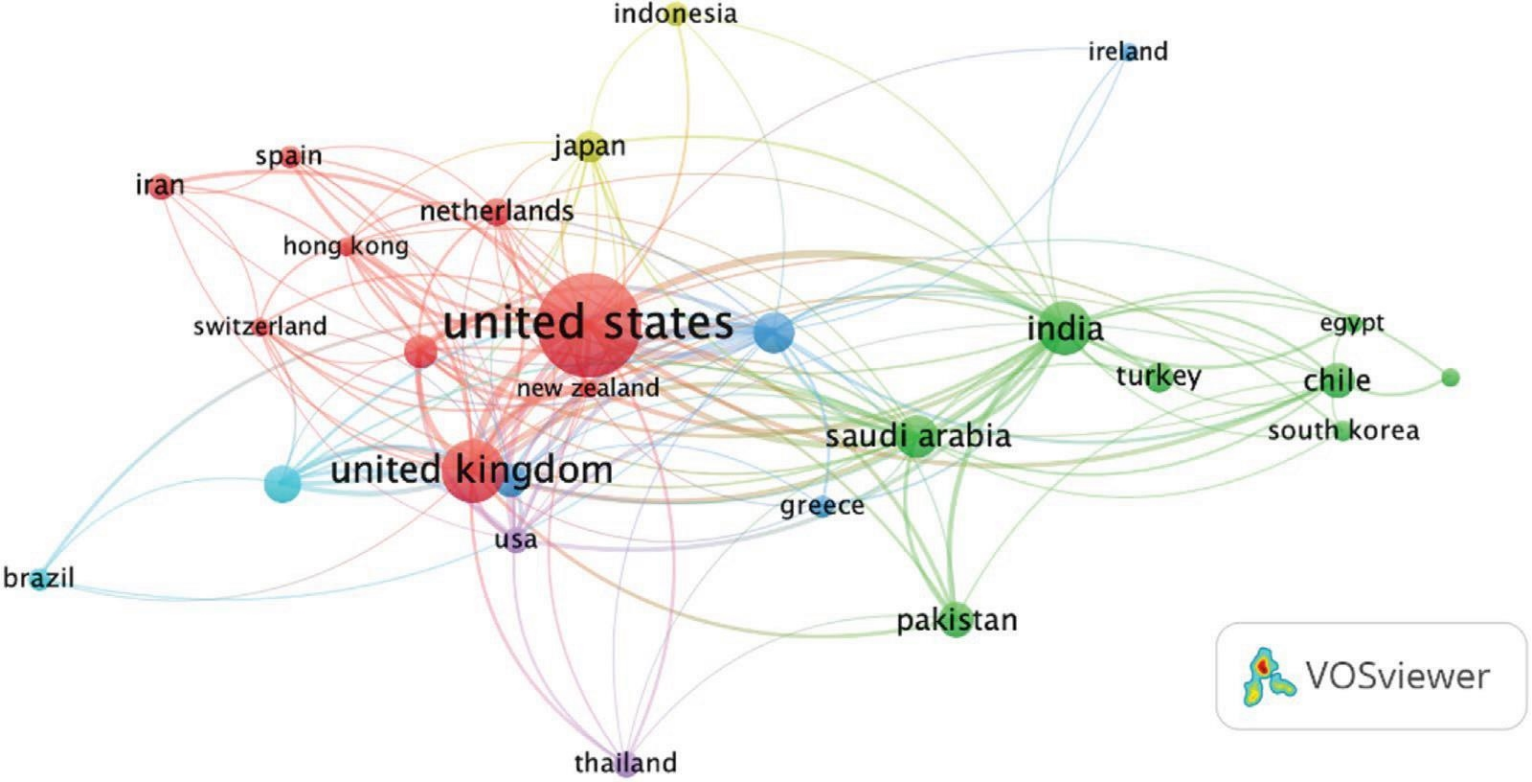
(B)

### 3.3. Thematic Analysis Uncovers Dominant and Neglected Skills

**Figure 4:**
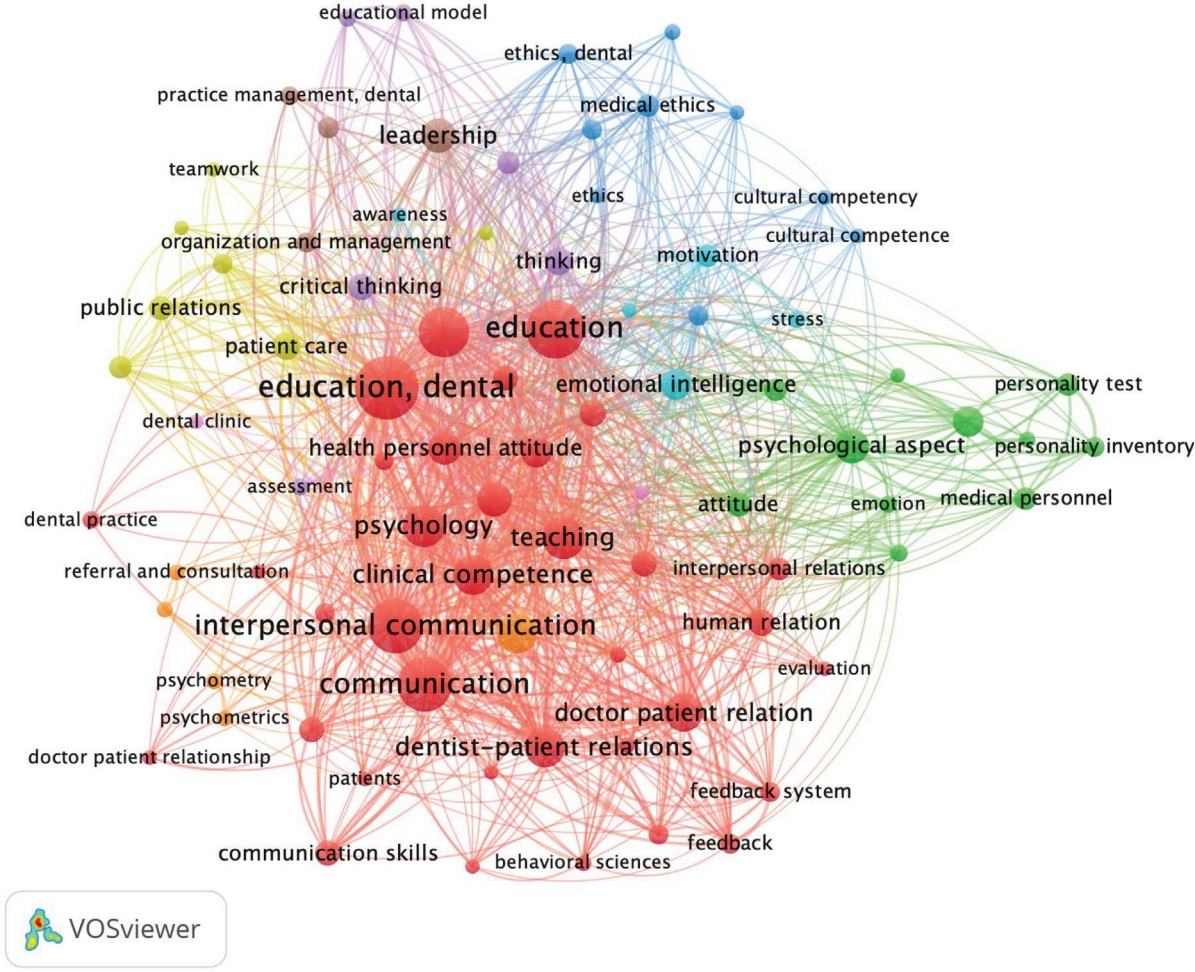
Keyword co‐occurrence network of non‐technical (soft) skills in dental education research. Mapping of 84 relevant keywords (threshold ≥ 10 occurrences) into nine thematic clusters, which can be grouped into five domains (1): professionalism and leadership (2), communication skills (3), personality and emotional intelligence (4), education and assessment and (5) behavioural sciences. Dense interconnections between communication skills and professionalism terms form the central hub, and while leadership, cultural competence and entrepreneurship occupy peripheral positions, reflecting thematic under‐representation.

Keyword co‐occurrence analysis of 2145 terms (threshold ≥ 10 occurrences) identified 173 meeting criteria; 84 were retained after relevance screening. A total of nine thematic clusters emerged, which can be grouped into five domains (Figure 4), each characterised by the following representative keywords:

*Professionalism and Leadership*: Example keywords: ‘professionalism’, ‘ethics’, ‘interprofessional education’, ‘teamwork’, ‘management’. This cluster dominated discussions on ethical practice and collaborative care, though leadership‐specific terms, for instance, ‘decision‐making,’ appeared less frequently.
*Communication Skills*: Example keywords: ‘patient interaction’, ‘empathy’, ‘interpersonal communication’. The most frequent theme (41.66% of keyword frequency) emphasised patient‐centred dialogue.
*Personality and Emotional Intelligence*: Example keywords: ‘psychological aspects’, ‘self‐awareness’, ‘emotional intelligence’. Highlighted traits critical for stress management and adaptability.
*Education and Assessment:* Example keywords: ‘curriculum’, ‘evaluation’, ‘critical thinking’, ‘feedback’, Focused on pedagogical strategies for non‐technical (soft) skill integration.
*Behavioural Sciences*: Example keywords: ‘clinical competence’, ‘doctor‐patient relationships’, ‘behavioural change’. Bridged psychological theories with practical clinical applications.

Alignment with citation trends revealed that communication skills were the most frequent domain, accounting for 41.66% of keyword frequency (Table 3). Professionalism and leadership appeared in 23.80% of keyword occurrences, while cultural competence accounted for <10%, indicating thematic under‐representation in these areas.

**Table 3:** The publications that had more than 50 citations.

Document	Citations
Carey JA, et al. Communications skills in dental education: a systematic research review. Eur J Dent Educ 2010;14(2):69‐78.	107
Hannah A, et al. A communication skills course for undergraduate dental students. J Dent Educ 2004;68(9):970‐7.	89
Gonzalez MAG, et al. Soft skills and dental education. Eur J Dent Educ 2013;17(2):73‐82.	64
Poole A, et al. Predicting performance in Canadian dental schools: the new CDA structured interview a new personality assessment, and the DAT. J Dent Educ 2007;71(5):664‐76.	64
Alzahem AM, et al. Stress management in dental students: a systematic review. Adv Med Educ Pract 2014;5:167‐76.	58
Victoroff KZ, et al. What is the relationship between emotional intelligence and dental student clinical performance? J Dent Educ 2013;77(4):416‐26.	58
Pau AKH, et al. Emotional intelligence and stress coping in dental undergraduates—a qualitative study. Br Dent J 2004;197(4):205‐9.	58
Hottel T, et al. Improvement in the interpersonal communication skills of dental students. J Dent Educ 2005;69(2):281‐4.	54
Hannah A, et al. Emotional intelligence and clinical interview performance of dental students. J Dent Educ 2009;73(9):1107‐17.	53
Broder HL, et al. Promoting interpersonal skills and cultural sensitivity among dental students. J Dent Educ 2006;70(4):409‐16.	53
Piazza‐waggoner CA, et al. Stress management for dental students performing their first pediatric restorative procedure. J Dent Educ 2003;67(5):542‐8.	50

### 3.4. Citation Analysis

The geographic distribution of citations revealed that publications from the United States had the most significant impact, followed by those from the United Kingdom, Canada and India. The network formed seven citation clusters, with North American, European and selected Asian countries exhibiting the strongest interconnectedness (Figure 3B).

Of the 546 publications, only 11 papers received more than 50 citations (Table 3). The two most cited articles are Carey et al., 107 citations [6], and Hannah et al., 89 citations [7]. Both addressed communication skills in dental education. Six of the 11 most‐cited studies focused on communication skills, while none addressed cultural competence, reaffirming the thematic imbalances identified in the keyword analysis.

### 3.5. Keyword Co‐Occurrence Patterns

The keyword co‐occurrence network (Figure 4) demonstrated dense interconnections between communication skills and professionalism‐related terms, forming the central hub of research activity. In contrast, terms relating to leadership, cultural competence and entrepreneurship occupied peripheral positions, reflecting their lower research visibility. Behavioural sciences formed a separate, well‐defined cluster linked to clinical competence, feedback mechanisms and the doctor–patient relationship. These findings reinforce the need for curriculum innovation in the underrepresented domains.

### 3.6. Curriculum Mapping and Alignment With Bibliometric Findings

The 2022 undergraduate dental curriculum incorporated targeted modules to address thematic gaps that can be mapped in the literature (Supporting Information 2: Table S2). The Supporting Information 2: Table S2 outlines courses across all six academic years and indicates the specific non‐technical (soft) skill competencies, such as communication, professionalism, leadership, critical thinking and teamwork, targeted in each course as part of the integrated curriculum reform.

i. Leadership and conflict resolution—introduced in Year 6 within Professional Development VI, responding to the low representation of leadership research.
ii. Interprofessional collaboration—strengthened through case‐based and team‐based learning activities to align with teamwork and professionalism clusters.
iii. Cultural sensitivity training—integrated into communication and behavioural science modules to address the representation of cultural competence in the literature.

Non‐technical (soft) skills training was embedded across all 6 years of the program:

i. Years 1–2: self‐awareness, ethics, stress management and foundational communication skills.
ii. Years 3–4: advanced communication, teamwork and leadership preparation for clinical years.
iii. Years 5–6: practice management, professional law, management and advanced leadership.

Assessment strategies included reflective writing portfolios, faculty formative feedback and targeted evaluations of behavioural science competencies.

In conclusion, the last decade has witnessed a substantial increase in publications on non‐technical (soft) skills in dental education, with a concentration in a small number of high‐impact journals. Research output is geographically skewed towards high‐income countries, with Africa and South America underrepresented. Communication skills and professionalism dominate the thematic landscape; leadership, cultural competence and entrepreneurship remain comparatively neglected. Bibliometric findings have been translated into structured curriculum reforms at Chulalongkorn University, integrating underrepresented competencies through targeted learning modules and assessment strategies.

## 4. Discussion

Our analysis underscores the growing acknowledgement of these skills as essential competencies in this field. The results reveal a dynamic research landscape characterised by thematic clusters, geographic disparities and evolving curricular integrations. By quantifying the foregoing elements, we provide a robust evidence base to guide curriculum reform and prioritise future research in this underrepresented domain. In the following sections, we contextualise our findings, highlight gaps and propose actionable insights for educators and policymakers.

### 4.1. Thematic Dominance and Under‐representation in Research

The bibliometric analysis identified five key domain clusters, with communication skills dominating in the literature, accounting for nearly 41.66%. This trend aligns with the global emphasis on patient‐centred care and interprofessional collaboration in healthcare. However, under‐representation of leadership and cultural competence/entrepreneurship signals a critical gap. While communication and professionalism are well‐integrated into curricula, as evidenced by their high citation rates, their breadth and practical application vary widely across institutions, as pointed out by Carey et al. [6] and Hannah et al. [7].

Evidence suggests that innovative teaching methods, such as simulated patients, video feedback, public speaking exercises and role‐playing, positively impact dental students’ communication competence [8–10]. Conversely, leadership and cultural competence remain poorly defined, reflecting historical curricular priorities that have focused primarily on clinical training. However, this also presents opportunities for innovation.

The case study from Chulalongkorn University illustrates how targeted modules in leadership and cultural sensitivity can effectively address these gaps, aligning with broader calls for holistic competency frameworks [1, 2].

### 4.2. Geographic Disparities and Collaborative Networks

Our findings reveal a geographic skew towards high‐income countries, particularly the United States and the United Kingdom, which contributed ~27% and ~10% of publications, respectively. This trend mirrors broader inequities in dental education research. Low‐resource regions, such as Africa and South America, accounted for <5% of output, despite facing unique healthcare challenges. Collaborative networks revealed strong partnerships among high‐output countries, such as the United States–Chile and the UK–India, but limited inclusion of underrepresented regions. This disparity underscores the need for capacity‐building initiatives and equitable research partnerships. For example, integrating non‐technical (soft) skills training in the resource‐constrained regions could leverage digital tools, such as virtual simulations, to bridge existing gaps, as suggested in recent studies [8, 11].

### 4.3. Curriculum Integration: Bridging Research and Practice

The exemplified case study of Chulalongkorn University’s 2022 curriculum reform demonstrates the mapping of those skills identified from bibliometric insights, thereby implying their practical application. The non‐technical skills content in the curriculum is mapped to those identified through bibliometric analysis to ensure comprehensive coverage of the skills. During curriculum development, a competency‐based approach was utilised. According to the Thai Dental Council Competency statement for the undergraduate curriculum, several non‐technical (soft) skills are listed, including moral and ethical values, lifelong learning, management and leadership, communication and self‐evaluation. These competencies are based on organising content, learning experience and assessment. These skills are aligned with those identified in the bibliometric analysis. The curriculum content addresses under‐representation, including leadership, conflict resolution, cultural sensitivity and entrepreneurship. These alignments indicate the coverage and comprehensive contents to foster non‐clinical (soft) skills for undergraduate dental students. However, it should be noted that assessing outcomes for these competencies can be challenging. Direct observation with structured rubric‐based assessment and self/peer‐assessment can be utilised to evaluate the demonstration of the desired behaviours. However, these methods cannot fully guarantee that such behaviours and skills have been successfully internalised or sustained over time. The curriculum has been implemented for only 3 years, and students are still progressing through the program. Consequently, the overall evaluation of non‐technical skill competencies remains limited.

By embedding non‐technical (soft) skills across all 6 years of study, from foundational communication in Years 1–2 to advanced leadership and practice management in Years 5–6, the curriculum addresses thematic gaps identified in the literature. For instance, the module of *Professional Development VI* in Year 6 of the curriculum incorporates leadership and management, addressing skills that are clearly underrepresented. Furthermore, the use of diverse pedagogical methods, such as role‐playing and reflective portfolios, further aligns with evidence suggesting that active learning enhances skill retention [9, 12]. This model, we believe, offers a transferable framework for other institutions, particularly in balancing non‐technical (soft) skills and hard skills and competencies.

Equally important is the integration of higher‐order thinking skills, such as critical thinking, problem‐solving and creative thinking, which underpin clinical judgement and adaptability. These skills can be assessed using validated tools, such as the HEIghten Critical Thinking Assessment, the Williams Critical Thinking Assessment or the Wagner Assessment Test [13, 14]. Implementing problem‐based learning and reflective problem‐solving approaches can develop students’ ability to analyse complex clinical scenarios, while fostering creative thinking, which supports innovation in patient care and treatment planning [15, 16]. Embedding these elements alongside leadership and teamwork modules creates a balanced, evidence‐informed competency framework for contemporary dental graduates.

### 4.4. Study Limitations

The current study has a few limitations. First, while Scopus database provides comprehensive coverage of high‐impact, English‐language journals, its under‐representation of regional and non‐English publications may introduce selection bias. This could lead to an incomplete portrayal of global research trends, particularly for studies from low‐ and middle‐income countries or those published in languages other than English. Consequently, the observed geographic and thematic imbalances in non‐technical (soft) skills research may partly reflect database limitations rather than true disparities in scholarly activity. Further, grey literature, trial registries, hand‐searching or citation chasing were not included in the methodology. Hence, relevant studies that were not published or indexed in the database could be missed, limiting the comprehensiveness of the findings. In addition, the searches were restricted to the article title field, rather than the article title, abstract and keywords field, to enhance the specificity and relevance of the identified publications. However, this approach could potentially lead to missing some relevant publications.

Limiting to a single database could introduce bias and miss relevant studies. To address this caveat in future studies, a multi‐database (e.g., SciELO, AJOL [Africa], CNKI [China]) search would be advantageous so as to enhance coverage of biomedical education literature to be more representative of culturally relevant research. Collaborations with regional dental education networks (e.g., SEAADE [Asia], ADEE [Europe]) could also uncover valuable unpublished curricular innovations, strengthening the evidence base for future bibliometric analyses.

The present study did not use the normalised citation matrix. Hence, older publications may appear influential and receive high citation counts due to accumulated citations. This limitation can bias the interpretation of the bibliometric analysis. Further, variations in keywords can lead to the omission or inflation of relevant topics and publications and, subsequently, bias in thematic clusters. With these limitations, the interpretation of this bibliometric analysis should be done with caution. Further detailed study is also needed to provide more precise information and potentially reduce bias.

The focus on a single‐institution case study, though illustrative, limits generalisability. The curriculum reforms at this premier Thai university reflect a specific institutional and cultural context and therefore necessitate validation in other settings, particularly those with different healthcare systems, educational resources or ecosystems. Future research should, therefore, include multi‐institutional collaborations to compare the integration of non‐technical across diverse geographic and socioeconomic contexts. Additionally, longitudinal studies tracking graduates’ patient outcomes, such as patient satisfaction or professional success, are warranted.

### 4.5. Challenges and Future Directions

Despite progress, challenges remain, including faculty readiness to teach and impart non‐technical (soft) skills, assessment variability and crowded curricula. Standardised assessment tools, such as the HEIghten Critical Thinking Assessment [14], could mitigate these challenges by providing measurable outcomes. Additionally, the bibliometric results highlight the need for further research on underrepresented skills, such as cultural competence and contextual adaptations in low‐resource settings. Future studies should also investigate the longitudinal impact of non‐technical (soft) skills training on patient outcomes, an area that is currently underrepresented in the literature.

## 5. Conclusion

Our bibliometric analysis of the pedagogy of non‐technical (soft) skills in dental education identifies significant thematic and geographic disparities. The accompanying case study presents a replicable framework for integrating underrepresented skills, such as leadership, into curricula. To address these imbalances, we recommend (1) targeted funding for research in underrepresented geographic regions, such as Africa and South America; (2) standardised tools to longitudinally track the impact of non‐technical (soft) skills on long‐ and short‐term patient care and (3) cross‐institutional partnerships to share best practices in curriculum design. By prioritising these actions, the dental education community can ensure that graduates are equipped not only with technical expertise but also with the essential interpersonal and cognitive competencies demanded by an evolving and challenging healthcare ecosystem.

## Author Contributions

Tanit Arunratanothai, Supachai Chuenjitwongsa, Piyamas Sumrejkanchanakij and Kanoknadda Tavedhikul contributed to data interpretation and critical manuscript revision. Thanaphum Osathanon contributed to conceptualisation, data acquisition, interpretation and manuscript drafting. Kausar Sadia Fakhruddin and Lakshman Samaranayake contributed to editing and critical manuscript revision and approval of the final version.

## Funding

The study is supported by the Faculty Development Fund, Faculty of Dentistry, Chulalongkorn University.

## Disclosure

After using the AI tool/service, the authors reviewed and edited the content as needed and took full responsibility for the publication’s content.

## Ethics Statement

The case study component of this research involved an analysis of literature from a public database and the analysis of existing curriculum documents at Chulalongkorn University, which did not require ethical approval as no human subjects or identifiable data were involved. All methods adhered to institutional guidelines for educational research.

## Conflicts of Interest

The authors declare no conflicts of interest.

## Supporting Information

Additional supporting information can be found online in the Supporting Information section.

## Supporting information


**Supporting Information 1** Table S1. Search strategy used for data acquisition in the Scopus database.

**Supporting Information 2** Table S2. Overview of Courses and associated non‐technical (soft) skill development objectives in the 2022 undergraduate dental curriculum (Year 1–Year 6) at the Faculty of Dentistry, Chulalongkorn University, Thailand.

## Data Availability

The data that support the findings of this study are available from the corresponding author upon reasonable request.

## References

[1] Kodali M. V. R. M. , Kodali U. S. , Gadicherla S. , Smriti K. , Singh A. , and Khurshid Z. , The Role of Soft Skills in Dental Education: Challenges and Importance, European Journal of Dentistry. (2025) 19, no. 3, 851–859, 10.1055/s-0044-1791938. 39657942 PMC12182425

[2] Gonzalez M. A. G. , Abu Kasim N. H. , and Naimie Z. , Soft Skills and Dental Education, European Journal of Dental Education. (2013) 17, no. 2, 73–82, 10.1111/eje.12017, 2-s2.0-84876335523. 23574183

[3] Elhaji A. , Omolade F.-A. , and Kashbour W. , Patient-Dentist Communication and Its Impact on Dental Services Utilisation as Perceived by Patients in Libya, Community Dental Health. (2024) 41, no. 1, 27–31, 10.1922/CDH_00260Elhaji05. 38373223

[4] Boonmak P. , Saensom D. , and Tangpukdee J. , et al.Perceptions and Influencing Factors of Interprofessional Collaboration in Final-Year Health Science Students, Journal of Interprofessional Care. (2024) 38, no. 6, 1109–1116, 10.1080/13561820.2024.2401363. 39365843

[5] van Eck N. J. , Waltman L. , Dekker R. , and Berg J. V. D. , A Comparison of two Techniques for Bibliometric Mapping: Multidimensional Scaling and VOS, Journal of the American Society for Information Science & Technology. (2010) 61, no. 12, 2405–2416, 10.1002/asi.21421, 2-s2.0-78449280370.

[6] Carey J. A. , Madill A. , and Manogue M. , Communications Skills in Dental Education: A Systematic Research Review, European Journal of Dental Education. (2010) 14, no. 2, 69–78, 10.1111/j.1600-0579.2009.00586.x, 2-s2.0-77953143775. 20522105

[7] Hannah A. , Millichamp C. J. , and Ayers K. M. S. , A Communication Skills Course for Undergraduate Dental Students, Journal of Dental Education. (2004) 68, no. 9, 970–977, 10.1002/j.0022-0337.2004.68.9.tb03846.x. 15342658

[8] Bock A. , Wagenknecht N. , and Winnand P. , et al.Improvement of Students’ Communication Skills Through Targeted Training and the use of Simulated Patients in Dental Education-a Prospective Cohort Study, BMC Medical Education. (2024) 24, no. 1, 10.1186/s12909-024-05818-z, 820. 39080578 PMC11290294

[9] Krause F. , Ziebolz D. , Rockenbauch K. , Haak R. , and Schmalz G. , A Video- and Feedback-Based Approach to Teaching Communication Skills in Undergraduate Clinical Dental Education: The Student Perspective, European Journal of Dental Education. (2022) 26, no. 1, 138–146, 10.1111/eje.12682. 33728768

[10] Hasan O. I. and Inglehart M. R. , Dental Hygiene and Dental Students’ Patient Communication Skills: Is Public Speaking Education Relevant?, Journal of Dental Education. (2025) 89, no. 7, 1051–1060, 10.1002/jdd.13799. 39707913 PMC12268115

[11] Zaug P. , Gros C. I. , and Wagner D. , et al.Development of an Innovative Educational Escape Game to Promote Teamwork in Dentistry, European Journal of Dental Education. (2022) 26, no. 1, 116–122, 10.1111/eje.12678. 33561894

[12] ADEA Commission on Change and Innovation in Dental Education , Hendricson W. D. , Andrieu S. C. , and Chadwick D. G. , et al.Educational Strategies Associated With Development of Problem-Solving, Critical Thinking, and Self-Directed Learning, Journal of Dental Education. (2006) 70, no. 9, 925–936, 10.1002/j.0022-0337.2006.70.9.tb04163.x. 16954414

[13] Anders P. L. , Stellrecht E. M. , Davis E. L. , and McCall W. D. , A Systematic Review of Critical Thinking Instruments for Use in Dental Education, Journal of Dental Education. (2019) 83, no. 4, 381–397, 10.21815/JDE.019.043, 2-s2.0-85064215234. 30745345

[14] Jauregui C. E. , Messer R. , Vitolo J. M. , and Young N. , Evaluating the use of HEIghten Critical Thinking Assessment to Monitor Critical Thinking in Dental Students, Journal of Dental Education. (2024) 88, no. 9, 1206–1212, 10.1002/jdd.13550. 38676393

[15] Byrne S. J. and Glasser S. , Creativity as a Framework for Innovation in Dental Education, Frontiers in Oral Health. (2023) 4, 10.3389/froh.2023.1233983, 1233983. 38024145 PMC10655018

[16] Kim Y. and Lee Y. H. , Creativity in Medical Education: Concepts Related to Creative Capacity, Yeungnam University Journal of Medicine. (2020) 37, no. 2, 79–83, 10.12701/yujm.2019.00458. 32146791 PMC7142024

