# Supplementary material for: Global Trends in Non‐Technical Skills Research in Dental Education: Bibliometric Review and a Curricular Case Study
Source: Int J Dent. 2026 Jan 26;2026:8633389. doi: 10.1155/ijod/8633389 (PMC12835188; doi:10.1155/ijod/8633389)
Supplement: Supplementary file 1 — Supporting Information 1 Table S1. Search strategy used for data acquisition in the Scopus database. [file IJOD-2026-8633389-s002.docx]

**Supplementary Table 1.** Search strategy used for data acquisition in the Scopus database

| **No.** | **Search Term** |
| --- | --- |
| 1 | (dentist* OR dental OR "oral health" OR stomatolog* OR odontolog* OR "oral medicine" OR orthdont* OR periodont* OR endodont* OR prosthdont* OR "oral implantolog*" OR "oral surg*" OR "oral hygien*" OR gerodont* OR caries OR cariology OR maxillofacial OR orofacial) |
| 2 | (("Education") OR ("Learning") OR ("Assessment") OR (“Academia”) OR (“Curriculum”) OR (“Gamification”) OR (“Training”) OR (“Mentoring”) OR (“Teaching”) OR (“Cognition”) OR (“Executive function”) OR (“Formative feedback”) OR (“Feedback”) OR (“Self assessment”) OR (“Problem-based”) OR (“Students”) or (“Study”) OR (“Summative feedback”) OR (“cognitive”) OR (“Evaluation”) OR (“Behaviourism”) OR (“Constructivism”) OR (“Cognitivism”) OR (“Connectivism”) OR (“Pedagogy”) OR (“Instructional Methods”) OR (“Student engagement”) OR (“Educational”) OR (“Professional Training”) OR (“Continuing education”) OR (“Competency-based education”) OR (“Active learning”) OR (“Team-based”) OR (“Case-based”) OR (“Experiential learning”) OR (“Blended learning”) OR (“Online learning”) OR (“E-learning”) OR (“Flipped classroom”) OR (“Simulation-based”) OR (“Peer learning”) OR (“Objective Structured Clinical Examination”) OR (“Rubrics”) OR (“Self-assessment”) OR (“Competency”) OR (“Virtual reality”) OR (“Augmented reality”) OR (“Learning management systems”) OR (“Digital learning”) OR (“Design Thinking”) OR (“Andragogy”) OR (“Instructional design”) OR (“Educational technology”) OR (“Distance learning”) OR (“Blended learning”) OR (“Collaborative learning”) OR (“Educational psychology”) OR (“Professional development”) OR (“Educational reform”) OR (“Lifelong learning”) OR (“Learning outcome*”)) |
| 3 | (("Soft skill*") OR ("Emotional intelligence") OR ("Social intelligence") OR (“Risk management") OR ("Communication”) OR (“Work ethics”) OR (“Professional ethics”) OR (“Leadership”) OR (“Active listening”) OR (“Conflict resolution”) OR (“Time management”) OR (“Creativity”) OR (“Adaptability”) OR (“Problem-Solving”) OR (“Interpersonal skill*”) OR (“Stress management”) OR (“Empathy”) OR (“Multitasking”) OR (“Honesty”) OR (“Negotiation”) OR (“Critical thinking”) OR (“Decision making skill*”) OR (“Personality”) OR (“Digital literacy”) OR (“Teamwork”) OR (“Leadership”) OR (“Risk management”) OR (“Practice management”) OR (“Personal Management”) OR (“Organisation administration”) OR (“priority management”) OR (“self-control”) OR (“Self-confidence”) OR (“Self-esteem”) OR (“Self-direction”) OR (“Self-confidence”) OR (“Goal setting”) OR (“Social expectation”) OR (“Responsibility”) OR (“Self-knowledge”) OR (“Self-respect”) OR (“Self-awareness”) OR (“Self-adaptation”) OR (“Emotional coping skill*”) OR (“Conceptual thinking”) OR (“Positive thinking”) OR (“System thinking”) OR (“Creative thinking”) OR (“Humanized”) OR (“Interprofessional communication”) OR (“Listening skill*”) OR (“Interprofessional collaboration”) OR (“Comparative thinking”) OR (“Logical thinking”)) |
| 4 | #1 AND #2 AND #3 PY = (2000/01/01-2025/02/ 09) AND LA=(English)and [Article or Review Article (Document Types)] |
