# Supplementary material for: Global Trends in Non‐Technical Skills Research in Dental Education: Bibliometric Review and a Curricular Case Study
Source: Int J Dent. 2026 Jan 26;2026:8633389. doi: 10.1155/ijod/8633389 (PMC12835188; doi:10.1155/ijod/8633389)
Supplement: Supplementary file 2 — Supporting Information 2 Table S2. Overview of Courses and associated non‐technical (soft) skill development objectives in the 2022 undergraduate dental curriculum (Year 1–Year 6) at the Faculty of Dentistry, Chulalongkorn University, Thailand. [file IJOD-2026-8633389-s001.docx]

**Supplementary Table 2**. Overview of Courses and associated non-technical (soft) skill development objectives in the 2022 undergraduate dental curriculum (Year 1-Year 6) at the Faculty of Dentistry, Chulalongkorn University, Thailand.

| **Years**** | **Courses** | **Related-Soft Skill Development Aspects** | **Domain Mapping** |
| --- | --- | --- | --- |
| Year 1 | Lifelong Learning Skills for Dentists | Lifelong Learning  Evidence-Based Learning  Critical Thinking  Design Thinking  Personal and Professional Development | *Professionalism and Leadership*  *Education and Assessment* |
|  | Basic Skills in Dentistry | Basic communication | *Communication Skills* |
|  | Skills in Integrated Biomedical Sciences and Bioethics | Critical Thinking  Technology Literacy  Morality and Ethics  Problem-Solving | *Professionalism and Leadership*  *Education and Assessment* |
|  | Professional Development I | Professionalism  Morality and Ethics  Thinking skills: Reasoning, Problem-solving, and Decision Making  Emotional and Stress Management  Personal and Professional Development  Communication  Patient-Dentist Interaction  Leadership and Teamwork  Information Management  Time Management  Digital Technology Literacy  Practice/Organization Management | *Professionalism and Leadership*  *Education and Assessment*  *Communication Skills*  *Behavioural Sciences*  *Personality and Emotional Intelligence* |
| Year 2 | Skills In Integrated Biomedical Sciences and Communication | Problem-Solving  Critical Thinking  Communication  Teamwork | *Education and Assessment*  *Communication Skills*  *Professionalism and Leadership* |
|  | Professional Development II | Professionalism  Morality and Ethics  Thinking skills: Reasoning, Problem-solving, and Decision Making  Emotional and Stress Management  Personal and Professional Development  Communication  Patient-Dentist Interaction  Leadership and Teamwork  Information Management  Time Management  Digital Technology Literacy  Practice/Organization Management | *Professionalism and Leadership*  *Education and Assessment*  *Communication Skills*  *Behavioural Sciences*  *Personality and Emotional Intelligence* |
| Year 3 | Clinical Skills in Dentistry | Clinical Communication | *Communication Skills* |
|  | Skills in Integrated Biomedical Sciences and Analytical Thinking | Problem-Solving  Critical Thinking  Analytical Thinking  Teamwork  Communication | *Professionalism and Leadership*  *Education and Assessment*  *Communication Skills* |
|  | Professional Development III | Professionalism  Morality and Ethics  Thinking skills: Reasoning, Problem-solving, and Decision Making  Emotional and Stress Management  Personal and Professional Development  Communication  Patient-Dentist Interaction  Leadership and Teamwork  Information Management  Time Management  Digital Technology Literacy  Practice/Organization Management | *Professionalism and Leadership*  *Education and Assessment*  *Communication Skills*  *Behavioural Sciences*  *Personality and Emotional Intelligence* |
|  | Dental Research Methodology | Life-Long Learning  Critical Thinking  Communication | *Education and Assessment*  *Communication Skills* |
| Year 4 | Integrated Biomedical Sciences and Oral Biology | Critical Thinking  Analytical Thinking  Communication  Teamwork | *Professionalism and Leadership*  *Education and Assessment*  *Communication Skills* |
|  | Team Practice Learning and Reflection I | Critical Thinking  Analytical Thinking  Problem-Solving  Communication  Teamwork | *Professionalism and Leadership*  *Education and Assessment*  *Communication Skills* |
|  | Professional Development IV | Professionalism  Morality and Ethics  Thinking skills: Reasoning, Problem-solving, and Decision Making  Emotional and Stress Management  Personal and Professional Development  Communication  Patient-Dentist Interaction  Leadership and Teamwork  Information Management  Time Management  Digital Technology Literacy  Practice/Organization Management | *Professionalism and Leadership*  *Education and Assessment*  *Communication Skills*  *Behavioural Sciences*  *Personality and Emotional Intelligence* |
|  | Dental Research Project I | Life-Long Learning  Critical Thinking  Problem-Solving  Communication  Teamwork | *Professionalism and Leadership*  *Education and Assessment*  *Communication Skills* |
| Year 5 | Professional Development V | Professionalism  Morality and Ethics  Thinking skills: Reasoning, Problem-solving, and Decision Making  Emotional and Stress Management  Personal and Professional Development  Communication  Patient-Dentist Interaction  Leadership and Teamwork  Information Management  Time Management  Digital Technology Literacy  Practice/Organization Management | *Professionalism and Leadership*  *Education and Assessment*  *Communication Skills*  *Behavioural Sciences*  *Personality and Emotional Intelligence* |
|  | Team Practice Learning and Reflection II | Critical Thinking  Analytical Thinking  Problem-Solving  Communication  Teamwork | *Professionalism and Leadership*  *Education and Assessment*  *Communication Skills* |
|  | Dental Research Project II | Life-Long Learning  Critical Thinking  Problem-Solving  Communication  Teamwork | *Professionalism and Leadership*  *Education and Assessment*  *Communication Skills* |
| Year 6 | Professional Development VI | Professionalism  Morality and Ethics  Thinking skills: Reasoning, Problem-solving, and Decision Making  Emotional and Stress Management  Personal and Professional Development  Communication  Patient-Dentist Interaction  Leadership and Teamwork  Information Management  Time Management  Digital Technology Literacy  Practice/Organization Management | *Professionalism and Leadership*  *Education and Assessment*  *Communication Skills*  *Behavioural Sciences*  *Personality and Emotional Intelligence* |
|  | Team Practice Learning and Reflection III | Critical Thinking  Analytical Thinking  Problem-Solving  Communication  Teamwork | *Professionalism and Leadership*  *Education and Assessment*  *Communication Skills* |

**The table outlines courses across all six academic years, indicating the specific non-technical skill competencies, such as communication, professionalism, leadership, critical thinking, and teamwork, targeted in each course as part of the integrated curriculum reform.
